# Investigating the Factors Associated with the Level of Expression of Estrogen and Progesterone Receptors in Patients Suffering from Colorectal Cancer

**DOI:** 10.1155/2021/4478155

**Published:** 2021-10-15

**Authors:** Saleheh Salehi far, Maryam Soltani, Mahmoud Zardast, Mohammad Reza Ghasemian Moghaddam

**Affiliations:** ^1^Student Research Committee, Birjand University of Medical Sciences, Birjand, Iran; ^2^Clinical Research Development Unit of Razi Hospital, Birjand University of Medical Sciences, Birjand, Iran; ^3^Department of Pathology, School of Medicine, Cardiovascular Diseases Research Center, Birjand University of Medical Sciences, Birjand, Iran; ^4^Department of Surgery, School of Medicine, Cardiovascular Diseases Research Center, Birjand University of Medical Sciences, Birjand, Iran; ^5^Clinical Research Development Unit of Imam Reza Hospital, Birjand University of Medical Sciences, Birjand, Iran

## Abstract

**Background:**

The present study was performed to investigate the factors related to the expression level of estrogen and progesterone receptor in patients with colorectal cancer. *Material and Methods*. This crosssectional study was performed on 54 patients suffering from colorectal cancer referring to Imam Reza Hospital in Birjand during 2018-2019. After the biopsy performed during surgery, the specimen was sent for immunohistochemistry, and the status of receptors was determined. Eventually, the data were analyzed by SPSS 22.

**Results:**

Out of the 54 patients studied, 64.8% were male. The mean age of the patients was 62.28 ± 14.03 years. The level of expression of beta-estrogen receptors and progesterone receptors had a significant relationship with age, consuming drugs of abuse, and familial history (*P* = 0.001). Also, the level of expression of estrogen and progesterone receptors of patients with a more advanced stage of cancer was significantly lower (*P* = 0.001).

**Conclusion:**

The extent of expression of estrogen and progesterone receptors affects the progression and prognosis of disease. Thus, through hormone therapy, a step can be taken to reduce the progression and even to treat colorectal cancer.

## 1. Introduction

### 1.1. Epidemiology Of Colorectal Cancer

Cancer is one of the important health plights and the third cause of mortality worldwide. Colorectal cancer is the third common cancer globally, claiming 10% of cancer mortality [1]. Universally, most cases of colorectal cancer occur in industrial countries, though in less-developed countries because of adopting the Western lifestyle, its prevalence is progressively increasing [2]. Research has shown that over the past 10 years, mortality because of colorectal cancer has grown in Asia. Japan has the highest rate of incidence of colorectal cancer especially in men, but since it is one of the first countries in implementing the screening program in year, the rate of mortality is lower in this country compared to Europe [1, 3]. In Iran, the standard incidence of this cancer has been reported 7 cases per every 100,000 persons, and it is the fourth common cancer. This cancer with mortality rate of 1.90 per 100,000 comprises about 13% of mortalities resulting from digestive system cancers and 5.3% of nonaccidental mortality causes in Iran [4]. The mortality caused by colorectal cancer is increasing in Iran, whereby there is a direct relationship between older age and mortality of this cancer [5].

### 1.2. The Level of Expression of Estrogen and Progesterone Receptors

Since the formation of women's health initiative in 1991 and based on several epidemiological studies, the ovarian steroids have been considered to be protective against cancer. Different studies in animal models show low risk of developing cancer in the presence of estrogens. Nevertheless, some studies show that once the disease develops, estrogens inhibit cellular proliferation, while some others indicate that they induce mitogenic effects. The oncogenic effects of estrogens have been widely examined in breast and ovarian cancer, but its function in colorectal cancer is poorly understood. Concerning the receptors, it has been found that beta-estrogen receptor is the dominant isoform in the colon, and its expression is lost during progression of colon cancer. This suggests that these can have a very significant role in progression of this disease. Recent studies on the tumor specimens of affected patients have indicated that overexpression is associated with better prognosis, and they support its role as a possible target for prevention from chemotherapy. The alpha-estrogen receptor expression is minimum in colon cells as well as in colon cancer cells. Thus, most studies show that the protective effects of estradiol in the colon cancer are undertaken by beta type [6]. In addition to the established effects of estrogens on colon tumorigenesis, progesterone (P4) has also been known as another ovarian steroid involved in this disease. Some studies have reported no expression of progesterone receptor (PR) in colon tumors. In addition, some studies have proposed artificial progestins as factors preventing colon cancer [7].

### 1.3. The Aim Of Study

Considering the importance of expression of estrogen and progesterone hormones with this disease, the present study was conducted to examine the factors associated with the level of expression of estrogen and progesterone receptors in patients suffering from colorectal cancer.

## 2. Material and Methods

### 2.1. Study Design and Setting

In this crosssectional study, all individuals who had colorectal cancer based on clinical symptoms as well as histopathology and underwent surgery plus colon resection in Imam Reza Hospital in Birjand from 5 April 2018 to 20 September 2019 were included. After acquiring ethics code from Birjand University of Medical Sciences (Ethics code: Ir.bums.Rec.1397.257) and receiving written informed consent form from the patients, demographic variables including age, gender, using opium, cigarette, hookah, and alcohol, history of urethrosigmoidostomy, familial history of colorectal cancer, history of having inflammatory bowel diseases, and colorectal cancer localization were completed based on a predesigned checklist.

The biopsy specimen taken during surgery was sent for immunohistochemistry, whereby the positivity or negativity as well as grade of receptors in the tissue were evaluated. Briefly, first primary slides and histological specimens were prepared as paraffin blocks in pathology center of Imam Reza Hospital in Birjand from the cancerous operated tissue and then assessed by a pathologist. Next, proper blocks and specimens were chosen for IHC staining, whereby some cuts were given with microtome from the relevant blocks and then transferred onto salinized slides for IHC staining. The specimens that did not have suitable target tissue in the reexamination were excluded from the study. Immunohistochemistry is a process for locating proteins in the cells of a tissue. In this method, using monoclonal antibodies, cell antigens are identified. In this plan, ER and PR markers on the colorectal cancer specimens were evaluated as follows: preparing a slide smeared with poly-L-lysine adhesive (which was already available and used accordingly), preparing 5-6 micron thin cuts of paraffin block, placing the slides for 15-20 min in 60°C autoclave for deparaffination, passing a basket containing the slides from four xylol containers for 30 min, passing basket containing slides from four alcohol containers 70, 80, 90, and 100, washing with distilled water (5 clean distilled water containers), placing the basket in microwave oven E7 at 95-98°C with 10X retrieval antigen solution in order to withdraw and sensitize the cell wall for 30-45 min, washing the basket of slides in two clean tested water containers, dragging Daku pen or a pen smeared with Vaseline in the specimens to fade the markers and solutions, pouring oxygenated water 3% on slides in a moist environment for 30 min, washing with clean distilled water, pouring primary antibody ER and PR as a drop on the slides in a moist and preferably rather warm environment for better reaction for 90 min, washing with clean distilled water, pouring secondary antibody HRP enhancer as one drop on the slides in a moist medium and preferably warm for better reaction for 30 min, washing with clean distilled water, pouring the next antibody poly vie plus mouse/rabbit HRP label (Biosystem) as one drop on the slides in a moist and preferably warm medium for better reaction for 30 min, washing with clean distilled water, pouring DAN chromogen (Biosystem, 50 *μ*L in 1 cc special dissolved buffer) as one drop on slides in the moist and preferably warm medium for better reaction in a dark environment for 30 min, washing with distilled water, pouring hematoxylin (Gill) as one drop for 1 min, washing with distilled water, immersing the slides in absolute alcohol as one-shot movement, drying slides, placing in three clean xylol containers, mounting and labelling the slides, and observing under microscope. After IHC staining, regarding the level of expression of estrogen receptor and progesterone receptors, 5 and 3 slides had not been stained well, respectively, and thus could not be evaluated. Considering investigation of the level of estrogen and progesterone receptors, 49 and 51 patients whose relevant information was available were used, respectively.

### 2.2. Statistical Analysis

The level of expression of estrogen receptor was categorized into three groups: less than 10%, 10-50%, and more than 50% [8]. Once collected, the data were introduced into SPSS 22 and analyzed using Chi-square test (Fisher exact test) at 5% significance level.

## 3. Results

### 3.1. Distribution of Clinical and Pathological Variable in Patients Suffering from Colorectal Cancer

Out of the 54 patients studied, 35 (64.8%) were male, and the rest were female. The mean age of the patients was 62.28 ± 14.03 years (30-86 years). Prevalence of cigarette smoking, waterpipe smoking, drugs of abuse, and alcohol consumption in the patients was reported 33.3%, 22.2%, 59.3%, and 3.7%, respectively. Further, 16.7% (*n* = 9) of the patients had inflammatory bowel disease; 13% (*n* = 7) had familial history of colorectal cancer, and only 2 (3.7%) had a history of radiation to the hip. None of the patients had Lynch syndrome, genetic diseases such as FAP, or history of proctosigmoidoscopy (Table 1).

### 3.2. Distribution of Clinical and Pathological Covariates in According to ER *β* and PR Expression

The findings indicated that there was a significant relationship between age of the patients suffering colorectal cancer and the level of expression of estrogen plus progesterone receptors (*P* = 0.001). The level of expression of estrogen receptor in most of the patients younger than 50 years was 10-50%, and in most patients 50 years of age and older than 50 years was less than 10%. In addition, most of the patients younger than 50 years showed progesterone receptor expression of above 50%, while the level of expression of progesterone receptor in most patients older than 50 years was less than 10%.

The findings also showed that there was a significant relationship between the level of expression of estrogen and progesterone receptors and consuming drugs of abuse among the colorectal cancer patients (*P* = 0.001). Out of 29 patients (59.2%) consuming drugs of abuse, 25 (86.2%) had low level of receptor expression and 4 (13.8%) had moderate levels of receptor expression. The level of expression of progesterone receptors was as follows: 17 (87.1%) as low, 3 (9.7%) as moderate, and 1 (3.2%) as high.

Based on the findings of the present study, there was a significant relationship between the level of expression of estrogen plus progesterone receptors and grade of cancer (*P* = 0.001); the patients in more advanced grades of colorectal cancer showed lower level of expression of estrogen and progesterone receptors. In addition, the findings showed that there was no significant relationship between gender, cigarette smoking, waterpipe smoking, and having inflammatory bowel disease and level of expression of estrogen and progesterone receptors in patients with colorectal cancer (*P* > 0.05) (Table 2).

## 4. Discussion

Steroid hormones play important physiological roles in the reproductive system, bone, cardiovascular system, and brain functioning [9–11]. Estrogen has oncogenic and tumor-stimulating roles in different tissues such as the breast, ovaries, uterus, prostate, and colon [12–15]. It has recently been shown that estrogen and progesterone receptors are expressed in normal mucus tissue, malignant colon tissue, and rectal tissue. In line with advances in diagnostic techniques, the role of estrogen and progesterone in tumorigenesis and incidence of colon cancer became more evident [16]. Nevertheless, some studies have shown low expression of progesterone in colon tumors and its absence of carcinogenic effects in animal studies [17]. The present study was performed to investigate the factors associated with the level of expression of estrogen and progesterone receptors in patients suffering from colorectal cancer. Based on results, there was a significant relationship between the age of patients with colorectal and the level of expression of estrogen plus progesterone receptors; the level of expression of estrogen receptor was 10-50% in most patients younger than 50 years and less than 10% in most patients 50 years of age and above. In addition, most patients younger than 50 years showed progesterone receptor expression above 50%, while the level of expression of progesterone receptor in most patients above 50 years was less than 10%. In the study by Oh et al., to examine the risk factors of breast cancer with regard to estrogen receptor, progesterone receptor, insulin-like growth factor, and expression of Ki67 in normal breast tissue in 2017, the median level of estrogen expression was significantly higher in older women (equal to or greater than 50 years) than among those younger than 50 years of age, but the expression of progesterone had no significant relationship with age [18]. Some studies have also indicated that in populations in which most or all women were in postmenopausal period, 3-7% positive estrogen cells and 12-29% positive progesterone cells were observed in the normal epithelium of the breast of women who did not have breast cancer [19–23]. In the study by Zhou et al., there was a correlation between the expression of estrogen in tumor tissues and age of patients, while there was no correlation between the expression of progesterone and age of patients [24].

The findings also showed that there was a significant relationship between the level of expression of estrogen as well as progesterone receptors and consuming drugs of abuse in patients suffering from colorectal cancer. In spite of many observational experimental studies, the role of prescribing opium substances on the immune system of human body and progression of cancers is unclear. One of the major limitations in investigating clinical benefits is the biological diversity of tumors. Most studies performed on the effect of opium drugs on the course of cancers have examined digestive system, lung, prostate, and breast cancers; according to investigations by the researchers of this paper, no studies have been done on the effect of opium substances on the course of cancers and expression of steroid hormones [25, 26].

Based on the findings of the present study, there was a significant relationship between the level of expression of estrogen plus progesterone receptors and grade of cancer; in patients with higher grades of colorectal cancer, the level of expression of estrogen as well as progesterone was lower. Nevertheless, there was no significant relationship between gender, cigarette smoking, waterpipe smoking, and having inflammatory bowel disease and the level of expression of estrogen plus progesterone receptors in patients suffering from colorectal cancer. In the study by Wenxi et al., who examined immunohistological expression of estrogen in 65 patients suffering from colorectal cancer, it was found that the level of positive expression of estrogen was significantly higher in tumor tissues compared to normal tissue. Also, expression of estrogen was independent of age and gender of patients, as well as the size, site, and grade of Ducke tumors [27]. Some studies have also reported that the immunohistological expression of estrogen in colorectal cancer is not associated with age of patient, gender, position of tumor, pathological type, histological type, or grade of disease [28, 29]. Although Zhou et al. showed that the quantitative levels of estrogen and progesterone expression were higher in cancerous tissues compared to normal tissues, and there was a positive correlation between the level of expression of estrogen as well as progesterone in tumor tissues; there was no correlation between the expression of estrogen or progesterone and gender, grade, position, and size of tumor [24]. In the study by Zavarhei et al., the expression of progesterone had a significant correlation with the size and grade of tumor [30]. In line with the results of the present study, in the study by Azizun-Nisa et al. conducted on patients suffering from breast cancer, it was found that higher grades of breast cancer disease were associated with lower level of expression of estrogen and progesterone, and this difference was significant [31]. Nguyen-Vu et al., examining the association between beta-estrogen receptor and colorectal cancer, concluded that the beta-estrogen receptor reduces metastasis of colorectal cancer through a new mechanism called miR-205-PROX1. This study showed that oncogenic prosperhomebox 1 (PROX1) is a potential target of Rim-205. Also, Er*β* and miR-205 would reduce colorectal cancer compared to nontumorous colon, whole PROX1 level increases. This was in line with the results of the present study regarding the association between estrogen receptor and decreased colorectal cancer [32].

In this study by Botteri et al. conducted on patients with colorectal cancer, it was observed that hormone therapy was associated with decreased risk of colorectal cancer especially in the advanced stages of the disease [33]. Based on the results of various studies, beta-estrogen receptor (ER*β*) regulates DNA repair, increases apoptosis, and reduces cell replication. Thus, activation of ER*β* can shrink the tumor and prevent its progression [34, 35].

## 5. Conclusion

Accordingly, relying on the results of the present study as well as other pieces of research in this regard, it can be stated that the expression of estrogen and progesterone receptors is associated with the progression and prognosis of the disease. Possibly, through hormone therapy, one could take a step in reducing the progression of colorectal cancer and even in treating it. It is also suggested to conduct studies with larger sample size in this regard.

## Figures and Tables

**Table 1:** Distribution of clinical and pathological variable in patients suffering from colorectal cancer.

Characteristic	*N* (%)	Characteristic	*N* (%)
Gender (male)	35 (64.8)	Using alcohol	2 (3.7)
Age (≥50) years	39 (72.2)	History of inflammatory bowel diseases	9 (16.7)
Using cigarette	18 (33.3)	Familial history of colorectal cancer	7 (13)
Using opium	32 (59.3)	History of urethrosigmoidostomy	2 (3.7)
Using hookah	12 (22.2)		
*Colorectal cancer localization*			
Cecum			2 (5.6)
Ascending colon			6 (11.1)
Sigmoid colon			3 (5.6)
Colon transverse			2 (3.7)
Splenic flexure			3 (5.6)
Descending colon			7 (13)
Sigmoid			14 (25.9)
Rectosigmoid			4 (7.4)
Rectum			12 (22.2)

**Table 2:** Distribution of clinical and pathological covariates in according to ER *β* and PR expression.

Characteristic	ER *β* expression			*P* value	PR expression			*P* value
	Low *N* (%)	Moderate *N* (%)	High *N* (%)		Low *N* (%)	Moderate *N* (%)	High *N* (%)	
Gender								
Male	22 (68.8)	8 (25)	2 (6.3)	0.499	23 (67.6)	7 (20.6)	4 (11.8)	0.774
Female	9 (52.9)	7 (41.2)	1 (5.9)		10 (58.8)	5 (29.4)	2 (11.8)	
Age (years)								
<50	4 (28.6)	7 (50)	3 (21.4)	0.001	4 (26.7)	5 (33.3)	6 (40)	0.001
≥50	27 (77.1)	8 (22.9)	0 (0)		29 (80.6)	7 (19.4)	0 (0)	
Using cigarette								
Yes	11 (68.8)	5 (31.2)	0 (0)	0.456	12 (66.7)	5 (27.8)	1 (5.6)	0.565
No	20 (60.6)	10 (30.3)	3 (9.1)		21 (63.6)	7 (21.2)	5 (15.2)	
Using opium								
Yes	25 (86.2)	4 (13.8)	0 (0)	0.001	27 (87.1)	3 (9.7)	1 (3.2)	0.001
No	6 (30)	11 (55)	3 (15)		6 (30)	9 (45)	5 (25)	
Using hookah								
Yes	8 (72.7)	2 (18.2)	1 (9.1)	0.571	8 (66.6)	2 (16.7)	2 (16.7)	0.725
No	23 (60.5)	13 (34.2)	2 (5.3)		25 (64.1)	10 (25.6)	4 (10.3)	
History of inflammatory bowel diseases								
Yes	4 (44.4)	4 (44.4)	1 (11.2)	0.364	4 (44.5)	2 (22.2)	3 (33.3)	0.091
No	27 (67.5)	11 (27.5)	2 (5)		29 (69)	10 (23.8)	3 (7.1)	
Familial history of colorectal cancer								
Yes	1 (14.3)	4 (57.1)	2 (28.6)	0.004	1 (14.3)	3 (42.9)	3 (42.9)	0.004
No	30 (71.4)	11 (26.2)	1 (2.4)		32 (72.7)	9 (20.5)	3 (6.8)	
Grade								
I	0 (0)	7 (77.8)	2 (22.2)	0.001	0 (0)	5 (50)	5 (50)	0.001
II	8 (53.3)	6 (40)	5 (50)		9 (56.2)	6 (37.5)	1 (4)	
III	23 (92)	2 (8)	0 (0)		24 (96)	1 (6.3)	0 (0)	

## Data Availability

The dataset collected and analysed during the current study are available from the corresponding author on reasonable request.
